# Evaluation of multiple linear regression function and generalized linear model types in estimating natural menopausal age: A cross-sectional study

**DOI:** 10.18502/ijrm.v20i5.11052

**Published:** 2022-06-08

**Authors:** Nasrin Sadeghi, Hosein Fallahzadeh, Maryam Dafei, Maryam Sadeghi, Masoud Mirzaei

**Affiliations:** ^1^Department of Biostatistics and Epidemiology, Faculty of Health, Shahid Sadoughi University of Medical Sciences, Yazd, Iran.; ^2^Research Center for Healthcare Data Modeling, Departments of Biostatistics and Epidemiology, School of Public Health, Shahid Sadoughi University of Medical Sciences, Yazd, Iran.; ^3^Research Center for Nursing and Midwifery Care, School of Nursing and Midwifery, Shahid Sadoughi University of Medical Sciences Yazd, Iran.; ^4^Faculty of Mathematical Sciences, Ferdowsi University of Mashhad, Mashhad, Iran.

**Keywords:** Menopause, Etiology, Statistics, Numerical data.

## Abstract

**Background:**

Since women spend about one-third of their lifespan in menopause, accurate prediction of the age of natural menopause and its effective parameters are crucial to increase women's life expectancy.

**Objective:**

This study aimed to compare the performance of generalized linear models (GLM) and the ordinary least squares (OLS) method in predicting the age of natural menopause in a large population of Iranian women.

**Materials and Methods:**

This cross-sectional study was conducted using data from the recruitment phase of the Shahedieh Cohort Study, Yazd, Iran. In total, 1251 women who had the experience of natural menopause were included. For modeling natural menopause, the multiple linear regression model was employed using the ordinary least squares method and GLMs. With the help of the Akaike information criterion, root-mean-square error (RMSE), and mean absolute error, the performance of regression models was measured.

**Results:**

The mean age of menopausal women was 49.1 ± 4.7 yr (95% CI: 48.8-49.3) with a median of 50 yr. The analysis showed similar Akaike criterion values for the multiple linear models with the OLS technique and the GLM with the Gaussian family. However, the RMSE and mean absolute error values were much lower in GLM. In all the models, education, history of salpingectomy, diabetes, cardiac ischemic, and depression were significantly associated with menopausal age.

**Conclusion:**

To predict the age of natural menopause in this study, the GLM with the Gaussian family and the log link function with reduced RMSE and mean absolute error can be a good alternative for modeling menopausal age.

## 1. Introduction

Natural menopause comes after a permanent cease of menstruation for 12 months with a decrease of blood estrogen levels and an increase of follicle-stimulating hormone, which results in cessation of ovarian follicular development (1-3). This natural transition occurs in almost all women without any pathological or physiological causes (2). Evidence shows that the age at natural menopause differed among different races, so the mean age of menopause was 51.4 and 49-50 yr in western and Asian women, respectively (3). According to the literature in previous studies, menopause is associated with factors such as socioeconomic status, physical activity, marital status, education level, fertility history, etc. However, various factors can affect the age of natural menopause across the world (2, 4). With the onset of menopause, biochemical and hormonal changes occur, such as decreased estrogen hormone levels that cause behavioral (5) and physical disorders (6, 7). The premature or late occurrence of these changes may bring about premature menopause or late-onset menopause increasing the risk of having certain diseases in women. According to previous studies, early menopause correlates with an increased mortality rate caused by cardiovascular diseases (8, 9) and osteoporosis (10). While late menopause increases the risk of ovarian (11, 12), uterus (13), and breast cancers (14, 15).

Since women spend about one-third of their lifespan in menopause, accurate prediction of the age of natural menopause and its effective parameters are crucial to increase women's life expectancy (3). In medical data analysis, it is not always possible to establish regression assumptions. For example, it is possible that the response variable does not have a normal distribution, and even with different transformations by the researcher, a normal distribution for the response variable is not obtained. In these cases, generalized linear models (GLM) can be used due to less sensitivity when predicting than establishing regression assumptions. To the best of our knowledge, very few studies applied GLM to determine menopause age. The GLM models formed in 3 steps. In the first step, a suitable distribution for the response variable is determined, which could be a member of the exponential family. For example, for variables with the continuous response, Gaussian, Gamma, and Gaussian inverse distributions could be included. The second step is about the formation of the systematic component. This component is created by the linear combination of the predictor variables. The third step included applying the link function to establish a relationship between the random and systematic components (16-18).

Since regression assumptions are important for the ordinary least squares (OLS) model, in the absence of these assumptions, a common method before GLM models was to use transformations to normalize the distribution of the variable y-response and the variance stability. Nonetheless, for most types of data, such as medical data, it is difficult to find a transformation that stabilizes the variance in addition to the ability to normalize the data. In such cases, the type of transformation for normalization is usually different from the best transformation for variance stability. Another advantage of the GLM model is that the selection of the link function is different from the selection of the random component in this model. In the GLM model, the link function does not need to stabilize the variance and normalize the data. The fitting process does not limit this choice to the normal distribution by increasing the probability of choosing the probability distribution for Y.

These advantages of GLM models can be important for data from medical studies because regression assumptions cannot be established by conventional methods in some cases, and prediction error increases if these assumptions are not established, and the OLS models are used (19).

This study aims to compare the performance of various GLMs and OLS methods in predicting the age of natural menopause in a large population of Iranian women. We also examined and compared GLM and OLS in estimating the natural menopausal age and its effective factors.

## 2. Materials and Methods 

In this cross-sectional study, the data of 1706 women who were experienced menopause, registered in the Shahedieh Cohort study, Yazd, Iran from April 2015 to September 2017 were extracted.

The Shahedieh cohort study is a part of the PERSIAN cohort. The cohort study was carried out in Shahedieh, Zarch, and Ashkezar cities of Yazd due to homogeneity among people regarding a limited number of immigrants and emigrants, homogenous ethnicity, and local cooperation.

After establishing equipping the cohort center, the human resources were employed, and the research officers' team was trained to collect the required information. To collect the research data, residents of Shahedieh City who had 35-70 yr of age were required to refer to the predetermined health centers. Later, the participants' information was collected using a standardized questionnaire. Further information about the standardized questionnaire and PERSIAN Cohort can be found in related research (20). The inclusion criterion of this study was the women aged 35-70 yr who experienced menopause (21). The exclusion criteria were as follow: women with other menopausal etiology including breastfeeding, hormonal disorders, surgeries like hysterectomy or oophorectomy (22) and the individuals who had answered the question “Was your menopause a natural one?” No. After excluding the above-mentioned participants, a total of 1251 women participated in the study.

In this study, independent variables on the menopausal age were measured considering the present variables in cohort questionnaires, which included demographic characteristics (e.g., age, education level, and marital status), fertility history, body mass index (BMI), waistline, individual habits, social and economic status, physical activity, employment status, and some chronic illnesses. The participants' age was calculated when their entrance to the research. Based on the participants' last educational degree, the level of education was categorized into 5 groups: 1) illiterate, 2) elementary, 3) secondary, 4) high school, and 5) academic.

Single participants were 1 in 1251, and only 8 widows were reported. Therefore, due to a very limited number of samples, the singles, widows, and divorcees were merged into 1 group resulting in 2 main categories of 1) married and 2) widow/divorcee/single.

The participants' fertility history included the natural menopause age, menarche age, age at the first pregnancy, intake of oral contraceptives, number of pregnancies, use of infertility-related medications, history of salpingectomy and infertility.

The natural menopausal age was defined as the person's age at her last menstrual period that occurred after 12 months of menstruation (without pregnancy, lactation, etc.), and the menarche age was the age at first menstruation. Other variables included the use of oral contraceptives, salpingectomy history, infertility history, and intake of infertility-related medications, which were evaluated using 2 levels of Yes/No. Unfortunately, no data was found in the cohort profile concerning the period of using oral contraceptives and the dosage of other related medications for infertility treatment. The questionnaire sufficed to only one yes/no question that asked them whether they had used the medications or not. Thus there was no opportunity to examine such data in the present study.

The participants' BMI was also obtained by dividing weight in kilograms by square meters of height in meters (kg/m^2^). This variable was categorized under 3 levels of 1) ≤ 24.99 kg/m^2^, 2) 25-29.99 kg/m^2^, 3) ≥ 30 kg/m^2^. The waistline was measured in cm individually. The socio-economic status questionnaire contained questions including the highest educational qualification, housing status, number of households, and home facilities. After gathering data, the participants were classified as weak, moderate, and strong about their socio-economic status using the clustering method. The international physical activity questionnaires were administered to assess each individual's physical activity. The validity and reliability of this questionnaire were confirmed in Iran. The multiples of the resting metabolic rate (MET) were measured according to the international questionnaire for each individual.

According to this questionnaire, the total energy gain ranging from 0-599 (MET-min/wk) shows poor physical activity, from 600-3000 (MET-min/wk) indicates moderate physical activity, and higher than 3000 (MET-min/wk) represents severe physical activity. Individual habits include 2 variables of drug use, hookah, which were investigated using Yes/No levels. Smoking cigarettes is considered improper in Iran and Islamic culture accordingly among the 1251 women, even less than 5 were reported as smokers. Since this included smoking hookah, we merged them all with hookah smokers in 1 group. The period and the amount of usage were not asked in the questionnaire.

Chronic diseases considered in this study included the history of diabetes, cardiac ischemic, thyroid, kidney stones, gallstones, rheumatism, chronic headache, and depression. Since menopause begins 3-5 yr before its occurrence, we studied individuals whose diseases were diagnosed at least 4 yr before menopausal age.

### Ethical considerations

The research proposal was approved by the Ethics Committee of School of Public Health, Shahid Sadoughi University of Medical Sciences, Yazd, Iran (Code: IR.SSU.SPH.REC.1397.066).

### Statistical analysis

Statistical analyses were performed using R software, version 3.6.2 (R Core Team, R Foundation for Statistical Computing, Vienna, Austria) and SPSS software version 24 (IBM Corporation, Armonk, NY, USA).

To compare the mean age at menopause among different levels of variables which were categorized in 2-groups and multi-groups; in cases where the age at menopause was normal at each level of the variable, the parametric *t* test and ANOVA test were carried out respectively, however, in cases where the age wasn't normal, the nonparametric Mann-Whitney U-test and Kruskal-Wallis test were carried out. The correlation between the quantitative variables and the menopausal age was measured using the Pearson correlation coefficient. A significance level of 5% is considered. Aiming to investigate the impact of probable influential variables on menopausal age, the multiple linear regression method was applied using OLS and GLMs.

Multiple linear regression using the OLS technique is common in predicting natural menopausal age. However, the GLM is more general than the linear model since, in GLM, the response variable can have abnormal distribution. Moreover, the mean response variable can have a non-linear relationship with the predictor variables. Linear regression is a special case of GLM. To the best of our knowledge, very few studies applied GLM to determine menopausal age. The GLM models were formed in 3 steps. In the first step, we identified a suitable distribution for the response variable, which could be a member of the exponential family. Since menopause is a positive and continuous variable, GLM models were applied in this study, such as Gaussian, Gamma, and Gaussian inverse distributions. The second step was about the formation of the systematic component. This component was formed by a linear combination of the predictor variables. The third step included applying the link function to establish a relationship between the random and systematic components. The systematic component can hold any real number, but the variable can accept specific numbers as a subset of real numbers. An appropriate link function selection allows the prediction range to be within the response variable range. The log link function is an efficient link function for non-negative data (16-18).

The classic presumptions of regression were examined to conduct the multiple linear regression method with the OLS technique. The normality of menopausal age was examined utilizing Skewness and Kurtosis measures. The variance inflation factor was applied to identify the correlation between variables. If the variance inflation factor were greater than 10, a coincidence would exist between the independent variables (23).

The performance of regression models was examined by comparing them to each other using the Akaike information criterion (AIC) and also the root-mean-square error (RMSE) and mean absolute error (MAE) for measuring the error amount.

The AIC shows the extent of data loss by applying the considered statistical model. The MAE is the MAE between the predicted and observed menopausal age, and finally. The RMSE is the root mean square error of the predicted and observed menopausal age difference (24-26).

## 3. Results 

This cross-sectional cohort study investigated 1251 women who experienced natural menopause. The mean age was 58.7 ± 6.3 yr (95% CI: 59.0-58.3). Furthermore, the mean natural menopause age was 49.1 ± 4.7 yr (95% CI: 48.8-49.3) with a median of 50 yr. According to the results of univariate analysis, mean natural menopause age was significant across the educational levels, salpingectomy, infertility, diabetes, cardiovascular diseases, depression.

Illiterate people have devoted the highest mean age of menopause. Also, a history of salpingectomy, diabetes, ischemic heart disease, depression, and a history of infertility was accompanied by an increase and decrease in mean age at menopause, respectively (Table I).

As the found results claimed, the age of menopause has a direct and significant relationship with the number of pregnancies and the waistline. To put it in other words, with an increase in the number of pregnancies or the waistline, the age of menopause increases (Table II).

In all regression models, education, history of salpingectomy, diabetes, cardiac ischemic, and depression were significantly associated with menopausal age (Table III).

In a multiple linear regression model with the OLS technique, the age at menopause for the participant with an educational level of secondary decreases by -3.58. Also, in cases where the people had a history of salpingectomy, diabetes, cardiac ischemic, and depression, the menopausal age increases by 0.65, 2.04, 2.62, and 1.03, respectively (Table III).

Table IV shows the performance measurement criteria of MAE, RMSE, and AIC in each model. According to table IV, the MAE and RMSE amounts were found to be remarkably higher in multiple linear models with the OLS technique compared to GLM models. The AIC of the multiple linear models was almost the same as for the GLM with the Gaussian family.

**Figure 1:**
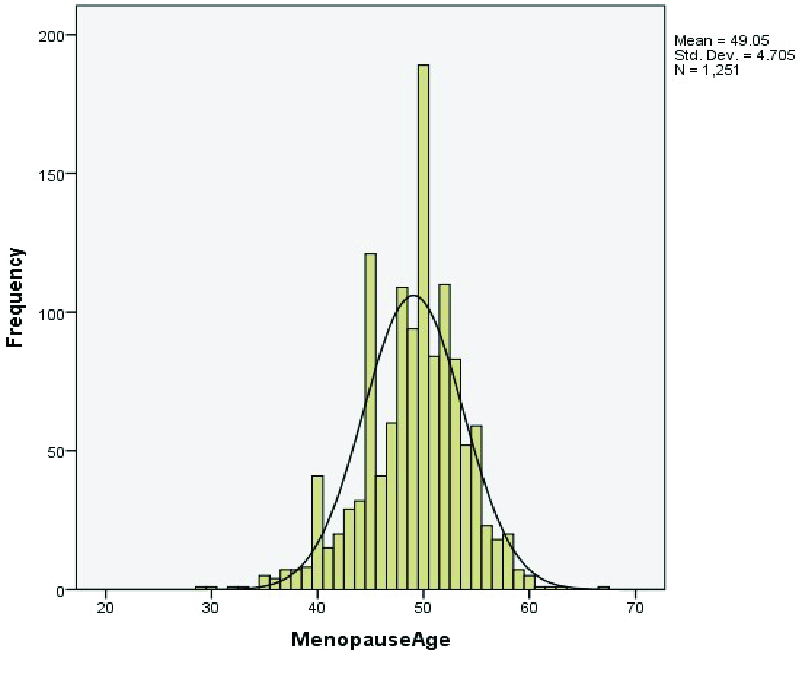
Distribution of age at natural menopause in 1251 studied women in Shahedieh city 2015-2017.

The distribution of menopausal age was illustrated in figure 1. The skewness and kurtosis of the natural menopausal age were from -2 to 2, which indicated the normal distribution of this variable.

**Table 1:** The association between menopausal age and demographic characteristics in study participants


**Variables**		**Mean ± SD**	**P-value**
Education level*			
	**Illiterate**	49.35 ± 4.78	
	**Elementary**	48.98 ± 4.23	
	**Guidance**	45.76 ± 5.28	
	**High school**	48.19 ± 5.72	
	**University**	46.50 ± 5.93	< 0.001
Marital status*			
	**Married**	49.00 ± 4.66		
	**Single, widowed, divorced**	49.34 ± 4.95		0.404
BMI*				
	**< 24.99**	48.34 ± 4.91	
	**25-29.99**	49.02 ± 4.49	
	**≥ 30**	49.28 ± 4.77	0.084
Socio-economic status*			
	**Low**	49.20 ± 4.97		
	**Moderate**	49.09 ± 4.40	
	**High**	48.14 ± 5.32	0.248
Have a job**		47.98 ± 4.90		0.053
Use of Hookah**		48.51 ± 6.02	0.78
History of drug use**		51.8 ± 3.38	0.058
History of salpingectomy**		49.56 ± 4.31	0.01
History of infertility**		47.74 ± 4.7	0.002
History of infertility medication**		47.9 ± 4.94	0.054
History of using contraceptive pills**		49.14 ± 4.31	0.79
Physical activity*			
	**Weakness**	49.39 ± 5.15	
	**Moderate**	49.19 ± 4.72	
	**High**	48.96 ± 4.69	0.73
History of diabetes**		51.23 ± 3.43		0.000
History of cardiac ischemic disease**		51.81 ± 4.78	0.004
History of thyroid disease**		49.15 ± 4.37	0.902
History of kidney stone**		48.55 ± 4.97	0.15
History of gallstone**		50.3 ± 4.25	0.11
History of rheumatic disease**		48.96 ± 5.16	0.94
History of chronic headache**		49.41 ± 4.35	0.6
History of depression**		50.07 ± 4.13	0.02
*KW: Kruskal-Wallis test, **MW: Mann-Whitney-u test, BMI: Body mass index, SD: Standard deviation			

**Table 2:** Pearson correlation coefficient between variables in women with natural menopausal age


**Variables**	**Mean ± SD**	**Correlation coefficient**	**P-value**
**Waistline**	100.78 ± 11.73	0.07	0.02
**Menarche age**	13.6 ± 1.59	0.026	0.365
**Number of pregnancies**	6.74 ± 3.17	0.09	0.001
**First pregnancy age**	18.51 ± 3.64	0.001	0.96
Pearson correlation test			

**Table 3:** Regression models for predicting age at natural menopause


		**Coefficient (B)**				**Standard error ( β )**				**P-value**			
		**GLM**			**GLM**			**GLM**		
**Variables**		**OLS**	**Gauss log**	**Gam log**	**Ivg log**	**OLS**	**Gauss log**	**Gam log**	**Ivg log**	**OLS**	**Gauss log**	**Gam log**	**Ivg log**
**Intercept**			45.40	3.80	3.82		3.82	2.40	0.05		0.66	0.66	0.00 ${}^{a}$	0.00 ${}^{a}$	0.00 ${}^{a}$	0.00 ${}^{a}$
**Education level **													
	**Illiterate**	Ref	Ref	Ref	Ref				
	**Elementary**	-0.40	-0.01	-0.40	-0.38	0.32	0.01	0.32	0.32	0.22	0.21	0.23	0.24
	**Guidance**	-3.60	-0.08	-1.03	-1.03	0.90	0.02	0.24	0.23	0.00 ${}^{a}$	0.00 ${}^{a}$	0.02 ${}^{a}$	0.01 ${}^{a}$
	**High school**	-1.70	-0.03	-0.50	-0.50	0.93	0.02	0.25	0.25	0.07	0.08	0.06	0.05
	**University**	-1.71	-0.04	-0.50	-0.50	1.50	0.03	0.40	0.39	0.24	0.25	0.24	0.24
**BMI**													
	**< 24.99**	Ref	Ref	Ref	Ref				
	**25-29.99**	0.51	0.01	0.14	0.14	0.45	0.01	0.45	0.46	0.26	0.26	0.25	0.24
	**≥ 30**	0.61	0.01	0.17	0.17	0.60	0.01	0.15	0.15	0.28	0.27	0.27	0.28
**Waistline**		0.01	0.00	0.05	0.05	0.10	0.00	0.06	0.06	0.46	0.47	0.45	0.44
**Marital status ref (married )**		-0.32	-0.01	-0.34	-0.36	0.40	0.01	0.39	0.40	0.40	0.42	0.38	0.36
**Employment status ref (no)**		-0.60	-0.01	-0.17	-0.18	0.60	0.01	0.16	0.16	0.32	0.33	0.29	0.27
**Use of hookah ref (no)**		-0.10	-0.002	-0.05	-0.09	0.75	0.02	0.20	0.20	0.91	0.88	0.94	0.97
**History of drug use ref (no)**		1.96	0.04	0.51	0.50	1.56	0.03	0.43	0.44	0.21	0.20	0.24	0.26
**Menarche age**		0.06	0.001	0.06	0.06	0.1	0.001	0.09	0.09	0.47	0.47	0.47	0.48
**Pregnancy n**		0.06	0.001	0.06	0.06	0.05	0.001	0.05	0.05	0.27	0.29	0.25	0.24
**First pregnancy age**		0.06	0.001	0.06	0.06	0.04	0.001	0.15	0.15	0.17	0.18	0.17	0.16
**History of salpingectomy ref (no)**		0.65	0.01	0.18	0.18	0.3	0.01	0.30	0.29	0.03	0.02	0.03	0.03
**History of infertility ref (no)**		-0.96	-0.02	-0.27	-0.27	0.63	0.01	0.20	0.17	0.13	0.13	0.12	0.11
**History of infertility medication** **use ref (no)**		-0.12	-0.003	-0.09	-0.06	0.85	0.02	0.23	0.23	0.89	0.86	0.92	0.94
**History of contraceptive drug ref (no)**		0.01	0.000	0.06	0.02	0.3	0.005	0.27	0.27	0.97	0.96	0.98	0.997
**Physical activity**													
	**Weakness**	Ref	Ref	Ref	Ref				
	**Moderate**	-0.2	-0.004	-0.13	-0.11	1.13	0.02	0.31	0.31	0.88	0.86	0.90	0.92
	**High**	-0.31	-0.007	-0.27	-0.24	1.12	0.02	0.31	0.31	0.78	0.75	0.80	0.83
**Socio-economic status**													
	**Low**	Ref						
	**Moderate**	0.07	0.001	0.07	0.08	0.30	0.01	0.30	0.30	0.82	0.82	0.81	0.8
	**High**	0.58	0.01	0.17	0.17	0.65	0.01	0.17	0.17	0.37	0.39	0.36	0.34
**History of diabetes ref (no)**		2.04	0.04	0.55	0.55	0.57	0.01	0.15	0.15	0.000 ${}^{a}$	0.000 ${}^{a}$	0.000 ${}^{a}$	0.000 ${}^{a}$
**History of cardiac ischemic ref (no)**		2.62	0.05	0.70	0.70	0.92	0.02	0.26	0.26	0.004 ${}^{a}$	0.003 ${}^{a}$	0.01 ${}^{a}$	0.01 ${}^{a}$
**History of thyroid diseaseref (no)**		-0.12	-0.002	-0.12	-0.13	0.60	0.01	0.16	0.16	0.84	0.85	0.84	0.83
**History of kidney stone ref (no)**		-0.99	-0.02	-0.27	-0.3	0.64	0.01	0.17	0.17	0.12	0.12	0.12	0.12
**History of gallstone ref (no)**		1.3	0.03	0.35	0.36	0.99	0.02	0.27	0.27	0.20	0.20	0.19	0.19
**History of rheumatic disease ref (no)**		-0.51	-0.01	-0.14	-0.15	0.92	0.02	0.25	0.25	0.58	0.60	0.57	0.56
**History of chronic headaches ref (no)**		0.43	0.01	0.46	0.48	0.4	0.01	0.4	0.4	0.3	0.3	0.26	0.24
**History of depression ref (no)**		1.03	0.02	0.28	0.28	0.44	0.01	0.45	0.45	0.02 ${}^{a}$	0.02 ${}^{a}$	0.02 ${}^{a}$	0.02 ${}^{a}$
OLS: Multiple linear regression model with OLS technique, GLM (Gauss log): Generalized linear model whit Gaussian family and log link, GLM (IG log): Generalized linear model whit inverse Gaussian family and log link, GLM (gam log): Generalized linear model whit gamma family and log link, BMI: Body mass index. ${}^{a}$ P < 0.05 is statistically significant													

**Table 4:** Performance measurement criteria


**Model**	**Mean**	**MAE**	**RMSE**	**AIC**
**OLS**	49.09 ± 1.21	3.45	4.46	7016.8
**GLM (family: Gaussian Link: log)**	49.09 ± 1.21	0.07	0.09	7017
**GLM (family: Gamma Link: log)**	49.09 ± 1.22	0.07	0.09	7060.4
**GLM (family: Inverse Gauss Link: log)**	49.09 ± 1.22	0.07	0.09	7090.9
OLS: Multiple linear regression model with OLS technique, GLM (family: Gaussian): Generalized linear model whit Gaussian family and log link, GLM (family: Inverse Gaussian): Generalized linear model whit inverse Gaussian family and log link, GLM (family: Gamma): Generalized linear model whit gamma family and log link. Mean: The average age of menopause predicted in each model. SD: Standard deviation from the age of menopause predicted in each model, AIC: Akaike information criterion, MAE: Mean absolute error, RMSE: Root mean squared error				

## 4. Discussion 

The mean and standard deviation of the menopausal age in our participants were 49 and 4.7 yr (95% CI: 48.8-49.3) and a median of 50 yr. The results of multivariable analysis showed that education, history of salpingectomy, diabetes, cardiac ischemic, and depression were significantly associated with menopausal age.

This mean was higher than the reported menopausal age of Yazd women (47.4) in 2007 (25). The achieved mean score for this region had been within the reported menopausal age range in Iran since 2004 (46.9-49.6) (3). Menopausal age was reported as 47.4, 48.6, 46.57, 48.3, and 49.6 yr in some cities of Iran, including Yazd, Isfahan, Tehran, Shiraz, and Hamadan, respectively (3, 25, 26). In general, menopausal age in Iran is lower than in developed countries due to socio-economic status and lifestyle factors (3). The univariate analysis using parametric and nonparametric tests showed that educational level, waistline, number of previous pregnancies, and the history of salpingectomy, infertility, diabetes, cardiac ischemic, and depression had a statistically significant effect on the menopausal age (p ≤ 0.05). The multiple linear regression model using the OLS technique showed that education at the secondary school level had a significant association with menopausal age. Since high education levels affect lifestyle, pregnancy health, and self-care, some studies indicated that menopausal age was higher in women with higher levels of education (27).

However, such a result was not found in the present study (25); we found that higher education had a negative coefficient in the regression equation. This may be due to the low number of higher education levels. Some studies confirmed the relationship between BMI and menopausal age (28, 29). But in the present study, this association was not significant, consistent with some previous studies in Iran. Furthermore no relationship was found between the waistline and menopausal age (3, 25). Multiple linear regression analysis showed no association between marital status and menopausal age. This was consistent with some studies (2, 3) and contradicted some others (4). According to studies, smoking and hookah have affected the factors such as gonadotropins and steroid hormones and leading to reduce follicle storage and ovarian aging, which is one of the causes of premature menopause (30). But in the present study, no association was found between smoking and hookah use and menopausal age. However, Yang and co-workers reported that smokers were at risk for early menopause (31). Several studies in Iran indicated that smoking was not associated with menopausal age. According to a study in Isfahan, this finding can be attributed to the fact that smoking is a taboo for women in the Islamic culture of Iran; therefore, women may not report it accurately (3, 25).

Furthermore, no relationship was observed between menopausal age and occupation; whereas, Koukouliata and colleagues confirmed this relationship (2). In multiple linear regression model, no association was found between menopausal age and reproductive history factors including menarche age, age at the first pregnancy (3), number of pregnancies, use of oral contraceptives, history of infertility, and use of infertility-related medications, but a significant and direct relationship was found between the history of salpingectomy and menopausal age. However, scarce information is available on the association of salpingectomy with menopausal age.

According to some studies results, regular exercise causes fitness and creates favorable conditions for reproduction and continuation. On the other hand, intense exercises lead to menstrual cycle problems. Hence, the exercise effect in most studies related to menopausal age is noticeable (32).

We observed no significant relationship between physical activity and menopausal age, which is similar to some studies in Iran (3). However, it was found that this relationship was significant over a population in Hamadan (33), which may be due to inaccurate reports of physical activity by participants in our study. The relationship between menopausal age estimation and its related factors in Isfahan was investigated, and it has been found that low socioeconomic status was significantly associated with early menopause (3). However, no significant relationship was observed between socio-economic status and menopausal age. One study have been conducted on some diseases and their impact on menopausal age. Given that menopausal symptoms occur 3-5 yr before menopause begins (34), we included those individuals whose diseases were diagnosed at least 4 yr before menopause. The studied diseases included diabetes, heart failure and angina, thyroid, kidney stones, gallstones, rheumatism, chronic headaches, and depression.

Based on multiple linear regression results, diabetes, cardiac ischemic, and depression were associated with aging in menopause, but some previous studies reported an inverse association between diabetes and menopausal age. In these studies, people with pre-menopausal diabetes were more likely to develop early menopause (35, 36). Considering that few studies were conducted on the relationship between chronic diseases and menopausal age, further studies are needed to establish the relationship between menopausal age and conditions. Likewise, the multiple linear models with the OLS technique, the education level, history of salpingectomy, diabetes, cardiac ischemic, and depression became significant in all GLM models.

The linear model is associated with many assumptions, which sometimes it is difficult or impossible to establish all of them. For example, predictor variables might interact or have a nonlinear relationship with the response variable, and even the response variable might not follow a normal distribution. In these cases, linear models are not necessary and optimal, and GLM models can be used.

A GLM is more general than a linear model because the response variable can have an abnormal or asymmetric distribution in this model. Moreover, the mean of the response variable can have a non-linear relationship with the predictor variables (16).

According to the obtained results, the multiple linear regression model with OLS technique and GLM model with the Gaussian family and the log link function have lower AIC than other models, which indicates their better performance. However, The GLM model with the Gaussian family and the log link function has much lower RMSE and MAE compared to the OLS multiple linear models.

The RMSE is a good criterion for measuring the model's prediction accuracy. If the aim is to predict a variable, it is one of the most essential criteria for model fit. The RMSE is measured in units of similar data, not in square units. The MAE is another criterion for measuring the performance of models. Like RMSE, MAE is also measured in similar units of data. Lower values of RMSE and MAE indicate better model fit.

In the discussion about poorer performance, it can be said that in the 2 models of gamma GLM and inverse Gaussian GLM rather than the OLS and Gaussian GLM models, since the above 2 distributions have better application for the response variable whose data set is continuous, non-negative and owing a positive skew, in the prediction of normal menopausal age, the response variable data set has almost a normal distribution, showing lower performance in this study.

Therefore, considering that the Gaussian GLM model has a better performance than the OLS model, it should be noted that in this model, the Gaussian distribution is a suitable distribution for variable response data sets and is less sensitive to regression assumptions.

Among the difficulties faced throughout the present study, one can note the lack of sufficient data in cohort questionnaire regarding some continuous variables such as the time length of oral contraceptive intake, the time length and the amount of hookah usage, the severity of chronic disease, and the control degree of the chronic diseases. Furthermore, some variables might not have been collected by the specialists after the menopause onset, and the data had been solely what the participant could recall at that time. This could result in deviations to predict the natural menopause age.

Also, in the present study, we were faced with limitations such as low sample size in some qualitative variables and absence of the mother's menopausal age variable.

## 5. Conclusion

In the present study, the mean and standard deviation of the menopausal age were 49 and 4.7 yr. Then to predict the normal menopause age, the GLM model with the Gaussian family and the log link function with decreasing RMSE and MAE can be a good alternative for modeling natural menopausal age and this model showed that education, history of salpingectomy, diabetes, cardiac ischemic, and depression were significantly associated with menopausal age.

##  Conflict of Interest 

The authors declare that there is no conflict of interest in this study.
